# Metastasis of Benign Leiomyomas Outside the Uterus

**Published:** 2018-05-18

**Authors:** Nathaniel A. Parker, Christopher S.R. Dakhil, Shaker R. Dakhil, Daniel Lalich

**Affiliations:** 1 Kansas City University of Medicine and Biosciences, Kansas City, MO; 2 Cancer Center of Kansas, Wichita, KS; 3 Wesley Medical Center, Wichita, KS

**Keywords:** leiomyoma, leiomyomatosis, uterine fibroids, gynecologic neoplasms

## Introduction

Uterine leiomyomas affect up to 30% of reproductive-aged women and they represent the most common gynecologic neoplasm in females.^1^,^2^ Diagnosis of classic uterine leiomyomata by radiology is not complex given their typical features on imaging and clinical manifestations. Ultimately, tissue diagnosis is definitive when leiomyoma is suspected on radiological imaging to distinguish it from leiomyosarcoma or other neosplasms. Leiomyomas are hormone-driven and most commonly arise from the uterus, but uncharacteristically can originate in the vulva, ovaries, bladder, and urethra.^3^ In addition, on rare occasions, they have been discovered in the tissues of bone, deep soft tissues, skin, mediastinum, skeletal and cardiac muscle, lymph nodes, omentum, mesentery, and retroperitoneum.^4^

Metastasis of uterine fibroids are rare events and have been given such names as benign metastasizing leiomyoma (BML), intravenous leiomyomatosis (IVL), disseminated peritoneal leiomyomatosis (DPL), retroperitoneal leiomyomatosis (RPL), and parasitic leiomyoma (PL).^3^ Multiple mysterious pulmonary nodules resulting from BML were first described by Marshall and Morris^5^ and Steiner.^6^ BML can be observed as a mass with histologically benign features, but also can demonstrate metastatic potential and present with diffuse lung tumors. This rare disease has gone by many titles**.** However, the current term, ‘benign metastizing leiomyoma’, represents a contraindication in the nomenclature. Steiner^6^ recommended the use of the term “metastasizing fibroleiomyoma”, as he thought the label of “benign” was incorrect.

Intravenous leiomyomatosis (IVL) is historically more rare than BML.^7^ Even more of an oddity, IVL can extend into the cardiopulmonary system including the right atrium, right ventricle, and pulmonary arteries. When this extension of these lesions into the cardiac system is present, IVL is termed intracardiac leiomyomatosis (ICLM). In 1974, ICLM was first reported in English.^8^ Likely due to technological advancements in imaging techniques, ICLM is being reported more often. However, definitive diagnosis is determined by postoperative pathological evaluation. IVL with cardiac extension is histologically benign. Thus, necrosis, mitoses, or cellular irregularities are rare.^9^

The tumors described above are typically benign entities, but uncommonly transition toward tissues of malignant potential. For two women who sought treatment for BML and IVL in our clinic, the primary goal was to outline and evaluate their specific genetic, pathological, and clinical features with the intention to elucidate possible treatment options.

## Case Presentations

### Case 1

A healthy, premenopausal, non-smoking, 30-year-old, Vietnamese woman with a negative family history of cancer was referred for multiple lung lesions. Two years prior, she underwent total hysterectomy for uterine fibroids. At that time, a single uterine fibroid was excised. Pathology confirmed a diagnosis of leiomyoma.

She initially presented with the chief complaints of flu-like symptoms and intermittent chest pain. Chest x-rays showed bilateral lung lesions. Bilateral breast mammograms showed no evidence of disease. Over the following years, the patient underwent an extensive outpatient workup, which included chest computed tomography (CT) scans and thoracoscopy, as well as bronchoscopy and bronchial washings, which proved to be unremarkable. Lung tissue biopsy was performed approximately nine years following the patient’s hysterectomy. Lung biopsies revealed benign-appearing smooth muscle nodules. Pathology showed a low mitotic index. These findings, along with the absence of coagulative necrosis and atypia, suggested the diagnosis of BML.

Over the course of the next two decades, the patient was evaluated by multiple specialists. Her lung lesions persisted despite exhaustive regiments, including preliminary progesterone therapy. Megestrol, tamoxifen, and medroxyprogesterone were added to her therapy regiment. No toxicities from these medications were observed.

She was referred to M.D. Anderson Cancer Center. Leuprolide and letrozole recommendations were made for her uterine leiomyoma metastasis of the lungs. No curative therapy was identified. Being that it was nine years post-hysterectomy, additional lung lesion tissue was acquired. Immunostaining revealed full negativity to c-kit and Her2/neu, but mixed negative epidermal growth factor receptor (EGFR) results with slightly positive staining of material among muscle cells and not the muscle cells themselves. More recently, genomic sequencing by Foundation® testing was performed with patient blood samples. An ALK: N1532D variant of unknown significance (VUS) was discovered. The patient was being conservatively managed with observation and symptom management.

### Case 2

A 33-year-old Caucasian woman of European descent presented to the emergency department with the chief complaint of abdominal pain. Ultrasound showed a large mass and follow-up CT scans revealed a pelvic mass. Surgical specialists performed a total hysterectomy and bilateral oophorectomy. At that time, a well-circumscribed mass was noted, solely adherent to the uterus and infundibulopelvic ligament. Pathology supported a diagnosis of leiomyoma. Serial sections of well-circumscribed right ovarian portions exhibited endometriotic cyst features, spindle cells without cytologic atypia, low mitotic activity, and absent malignant features. Spindle cell stromal growth predominated with similarities to fibromatosis (not otherwise specified).

Over time, the patient developed lung, cardiac, and intra-abdominal masses. Subsequent exploratory procedures revealed further gynecological tissue involvement, as well as retroperitoneal and extensive caval thrombus that extended bi-directionally into iliac veins and right atrium. At that time, approximately ten years post-hysterectomy, needle core biopsy of the intra-abdominal mass determined Müllerian origins, with benign smooth muscle features on histology. Immunostains of the tissue were negative for CD10, inhibin, S100, CD34, CD17, pancytokeratin CK7, and RCC. Desmin, actin, estrogen receptor, and WT-1 reacted positively, supporting a Müllerian origin. Emergent surgical intervention was considered.

Ultimately, caval thrombus mobilization, inferior vena cava reconstruction, total tricuspid porcine valve replacement, bipolar epicardial ventricular lead device placement (BEV), right radical nephrectomy, and removal of several large perirenal and retroperitoneal masses (> 5 cm) were performed. Samples from surgery were taken in all major areas. All tissue samples showed tumors made of spindle cells and absent necrosis and mitotic events. Ki67 testing showed a proliferation rate of less than 1%. Tissues with a vascular background were positive for CD34 (i.e., right atrium). Considering the patient’s history, presentation, tissue histopathology, and symptom progressions, the etiology of her disease was determined to be most likely due to IVL. After the patient’s multiple surgical interventions and stability were confirmed, she was discharged and followed as an outpatient. Recently, the patient’s tumor tissue specimens were analyzed with the genomic sequencing assay Foundation® to determine if the etiology of this condition could be due to somatic and/or environmental mutations. Testing identified three variants of unknown significance: EGFR:V674I, ERBB4:K1002R, and TSC2:L826M.

## Discussion

### Pathogenesis and Clinical Findings

Uterine leiomyomas are the most common gynecological tumor in women of reproductive age.^1^,^2^ These tumors typically are benign entities but uncommonly can make transitions toward tissues of malignant potential. Rare growth patterns of uterine leiomyomata have been observed, which include BML, IVL, DPL, RPL, and PL.^3^ The pathogenesis of these rare growth patterns is unclear. Furthermore, it remains uncertain which atypical growth pattern predominates over the others, due to all of their individual rarity. Based on the literature, BML, DPL and RPL has been observed to be more indolent.^3^, ^10^–^13^

In contrast, IVL has more aggressive characteristics.^9^ The most common findings in patients diagnosed with BML are single or multiple subcentimetric lung nodules with or without a concomitant diagnosis of uterine fibroids, DPL, or IVL. The pathogenesis of BML is remains unclear. However, it has been postulated BML spreads hematogenously, originates from independent multiple foci, and/or is hormone-driven.^3^, ^10^–^13^ Other atypical growth patterns that have been found to be hormone-driven include DPL, RPL, and PL. IVL has the unique feature of being incompletely hormone-driven. Clinical and pathological findings characteristic of IVL consist of in intraluminal growth in uterine and/or systemic veins, cord-like vessel lesions, and intracardiac extension, and tricuspid valve insufficiency.^3^, ^10^–^13^

The pathogenesis of these atypical growth patterns of uterine leiomyoma is unclear and controversial. These growth patterns are the result of clonal expansion of smooth muscle cells of the uterus, without significant cellular atypia or high mitotic index.^14^, ^15^ In contrast, leiomyosarcomas frequently exhibit the higher turnover rates and atypia. In addition, they rarely are seen with frequency rates of 0.1 to 6%.^16^ Primary lesions from these atypical growth patterns could be low-grade, slow growing leiomyosarcomas with inherently intact metastatic potential.^17^ Also, erroneous sampling falsely could support the diagnosis of benignity.^18^ However, recent cytogenetic studies have refuted this claim by showing that in contrast to leiomyosarcomas, these lesions have identical X-chromosome inactivation and a balanced karyotype.^19^,^20^

Metastasis of uterine fibroids most commonly appear several years after the diagnosis and removal of uterine leiomyomata by hysterectomy. Thus, prior gynecological surgery such as hysterectomy or myomectomy is a risk factor for developing any of these rare growth patterns that originate from benign uterine fibroids. In our two cases, the interval between uterine fibroid diagnosis, hysterectomy, and metastasis was two and zero years, respectively. Based on the review of literature, IVL appears to be less common phenomenon. An earlier report reviewing ten cases of BML observed an interval range of four to 23 years (mean 14.9 years) from the time of therapeutic hysterectomy to BML diagnosis.^17^ The data remained consistent with preceding case review work that described the interval between hysterectomy to BML diagnosis that ranged from three to 20 years (mean 10 years).^6^

### Genomic Sequencing

Genomic sequencing by Foundation® testing of blood and tissue samples from the patients in Cases 1 and 2 identified no genomic mutations in any currently established cancer-related gene. However, one variant of unknown significance (VUS) was detected in Case 1, ALK:N1532D, and three were detected in Case 2, EGFR:V674I, ERBB4:K1002R, and TSC2:L826M. In all samples from case patients, microsatellite status was determined to be stable and the overall tumor mutation burden was low (0.80 mutations/Mb).

These variants are termed as such because their alterations may not have been characterized adequately in the scientific literature at the time genomic sequencing was performed and/or the genomic context of these variants remains unclear. Thus, their clinical significance can neither be supported nor denied. Heightened VUS awareness poses a challenge to physicians not only for determining their relevancy, but for effectively communicating their importance to patients. Recent efforts have been made by National Center for Biotechnology Information (NCBI) to track and catalogue newly discovered variants with clinically relevant phenotypes.^21^ We postulate that these variants could be targeted as treatment options in the future when patients have failed all other previous therapies.

### Treatment

The benign versus malignant potential of BML and IVL remains unclear. Currently, there are no definitive guidelines regarding management due to their rare nature. BML is not only reliant on estrogen and progesterone, but also the majority of BML tumors are ER positive.^22^ GnRH analogs have been successful in treating BML.^23^,^24^ Progesterone antagonists have been discussed as possible adjuvant therapy for BML patients, but certain investigators advise against the use of these agents, at least not alone, because of their ability to up-regulate estrogen receptors.^25^ Rivera et al.^26^ believed anastrozole and raloxifene combination therapy could be as effective as the more traditionally used GnRH agonists and progesterone, even in postmenopausal patients with BML. Based on BML’s close relationship to uterine leiomyomas, some investigators are optimistic raloxifene could be a suitable treatment option for BML. The literature is unclear on the effectiveness of tamoxifen on BML lesions.^23^,^26^

IVL with cardiac extension, such as with the patient in Case 2, has been described in the literature and termed intracardiac leiomyomatosis (ICLM).^8^ ICLM is histologically benign. However, ICLM is suggested to be clinically aggressive due to the risk of sudden death caused by total outflow tract obstruction. Complete removal is the recommended treatment. Neoadjuvant and adjuvant anti-estrogen regiments or radiation therapy alone have not been shown to be a curative solution. This is due to the historical nature of IVL tumors to be incompletely hormone-driven. Finally, incomplete removal is not recommended due to previous studies reporting a near 30% recurrence rate if complete removal is not performed.^9^
